# Intrinsically p-type cuprous iodide semiconductor for hybrid light-emitting diodes

**DOI:** 10.1038/s41598-020-61021-2

**Published:** 2020-03-04

**Authors:** D. Ahn, J. D. Song, S. S. Kang, J. Y. Lim, S. H. Yang, S. Ko, S. H. Park, S. J. Park, D. S. Kim, H. J. Chang, Joonyeon Chang

**Affiliations:** 1 Peta Lux Inc., 3F TLi Building, 12 Yanghyeon-ro, 405 beon-gil, Jungwon-gu, Seongnam-si, Gyeonggi-do 13438 Republic of Korea; 2 Department of Electrical and Computer Engineering and Center for Quantum Information Processing, University of Seoul, 163 Seoulsiripdae-ro, Dongdaemun-gu, Seoul 02504 Republic of Korea; 3 Physics Department, Charles E. Schmidt College of Science, Florida Atlantic University, 777 Glades Road, Boca Raton, FL 33431-0991 USA; 4 Post-Silicon Semiconductor Institute, Korea Institute of Science and Technology Hwarang-ro 14 gil, Seoungbuk-ku, Seoul 02792 Republic of Korea; 5 Department of Physics, Kyung Hee University, 26 Kyungheedae-ro, Dongdaemun-gu, Seoul 02447 Republic of Korea; 6 Electronics Department, Catholic University of Daegu, 13 Hayang-Ro, Hayang-Eup, Gyeongsan-si, Gyeongbuk 38430 Republic of Korea; 7 WONIK IPS, 75 Jinwisandan-ro, Jinwi-myeon, Pyeingtaek-si, Gyeonggi-do 17709 Republic of Korea; 8 TLi Inc., 10 F TLi Building, 12 Yanghyeon-ro, 405 beon-gil, Jungwon-gu, Seongnam-si, Gyeonggi-do 13438 Republic of Korea; 9 Department of Materials Science & Engineering, Yonsei University, 50 Yonsei-ro, Seodaemun-gu, Seoul 03722 Republic of Korea; 10 Yonsei-KIST Convergence Research Institute, 50 Yonsei-ro, Seodaemun-gu, Seoul 03722 Republic of Korea

**Keywords:** Inorganic LEDs, Inorganic LEDs

## Abstract

Cuprous halides, characterized by a direct wide band-gap and a good lattice matching with Si, is an intrinsic p-type I-VII compound semiconductor. It shows remarkable optoelectronic properties, including a large exciton binding energy at room temperature and a very small piezoelectric coefficient. The major obstacle to its application is the difficulty in growing a single-crystal epitaxial film of cuprous halides. We first demonstrate the single crystal epitaxy of high quality cuprous iodide (CuI) film grown on Si and sapphire substrates by molecular beam epitaxy. Enhanced photoluminescence on the order of magnitude larger than that of GaN and continuous-wave optically pumped lasing were found in MBE grown CuI film. The intrinsic p-type characteristics of CuI were confirmed using an n-AlGaN/p-CuI junction that emits blue light. The discovery will provide an alternative way towards highly efficient optoelectronic devices compatible with both Si and III-nitride technologies.

## Introduction

**Figure 1:**
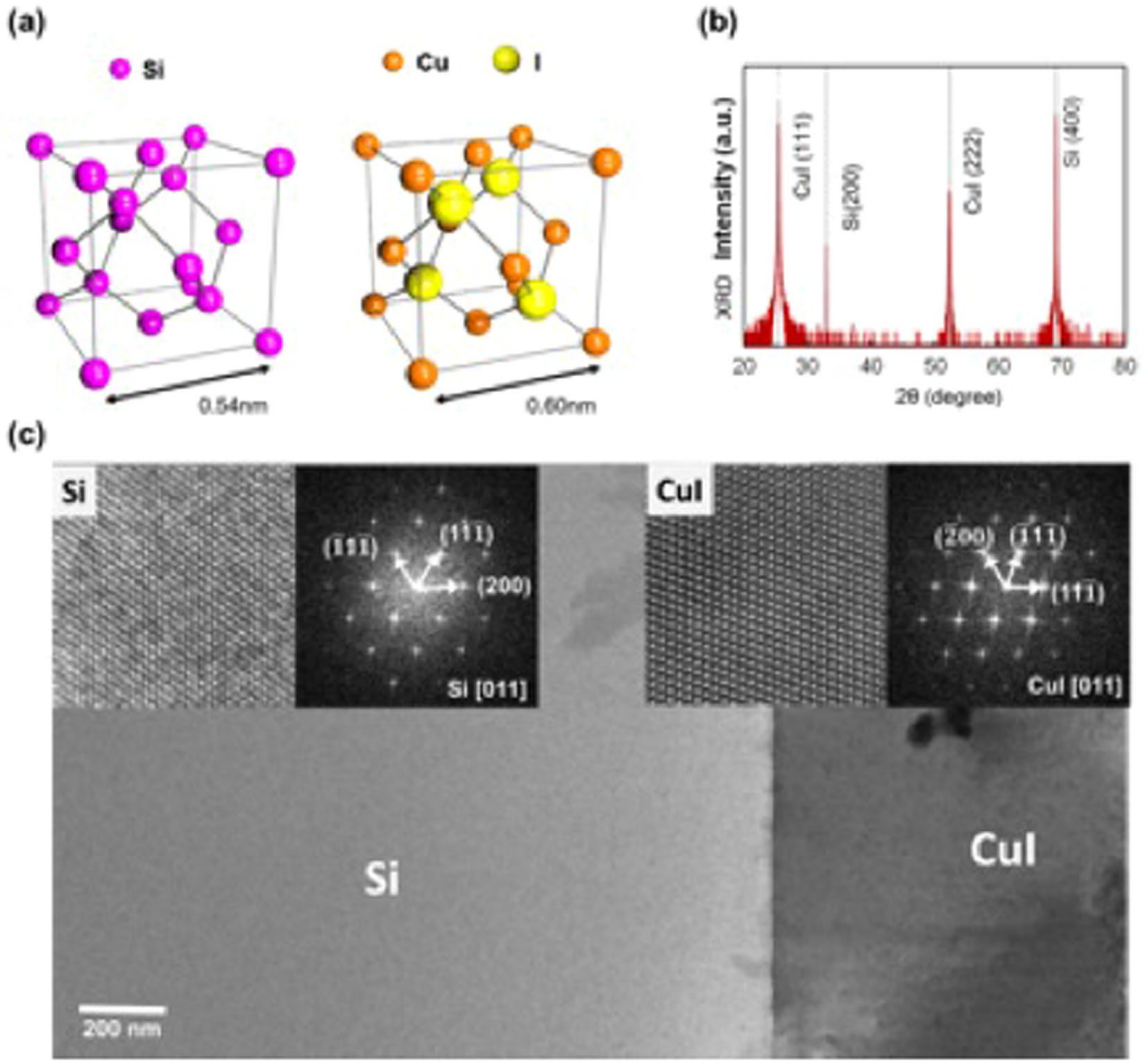
(**a**) Schematic diagram of the crystal structure for Si and CuI. Si is the diamond-crystal type lattice characterized by four covalently bonded Si atoms. The zinc blende lattice of cuprous halides crystals such as CuCl, CuBr and CuI consists of two interpenetrating face-centered cubic (fcc) lattices displaced along the diagonal of the body. Cu atoms on one fcc lattice and halogen atoms on the other side form the isomorphic structure minimizing the electrical polarization. (**b**) X-ray diffraction (XRD) 2θ scan of the CuI thin film (sample #3) grown on a Si substrate. The XRD peaks of CuI correspond to the (111) and (222) planes. (**c**) Transmission electron microscopy (TEM) image of the single crystalline structure of CuI grown on Si substrate. High resolution images and corresponding FFTs in the inset are obtained from each section of Si substrate and CuI film.

Cuprous halide atoms form a tetrahedrally coordinated isomorphic structure with the diamond-type lattice^1–16^. The Cu atoms are located on a face-centered cubic lattice, and the four nearest neighboring sites are occupied by halogen-type atoms, as shown in Fig. 1(a), which forms a zinc blende structure^8,17–26^. The zinc blende structure minimizes the piezoelectric effect resulting from polarization because it has four symmetry-equivalent polar axes and each electrical field contribution cancels each other. A semiconductor is polarized if it has a singular polar axis as in the case of the [0001] axis for wurtzite semiconductors, such as gallium nitride (GaN) and zinc oxide (ZnO)^27–35^. It is well known that the luminous efficiency of wurtzite structure is significantly affected by the presence of polarization. Large built-in electrostatic fields of the order of MV/cm in the active layer will result in long radiative lifetimes^28–35^. Since the piezoelectric effect of cuprous halides is much smaller than that of III-nitrides, we can easily ignore piezoelectric field effects for cuprous halide semiconductors, which is a great advantage for optoelectronic applications^1,23^.

Moreover, the large lattice mismatch between the nitride semiconductor and the commercially available substrates, typically sapphire or SiC, inevitably generates a high misfit dislocation density in the order of 10^10^ cm^−2^. The performance and reliability of the device would be degraded. Although a lot of epitaxial growth technologies have been developed to minimize misfit dislocation density, it is still suffered from the high density of defects which prevents realization of high performance electronic and optical devices on nitride semiconductor^2,4,16,20^. It would be desirable to have a kind of semiconductor with good lattice match to Si for the purpose.

Materials with high exciton binding energies have been studied in the hope of improving quantum efficiencies^36–42^. The exciton binding energy is regarded as a measure of the interaction between electrons and holes. It is used to predict the strength of electron-hole recombination processes that are related to the quantum efficiencies of light-emitting devices^41,42^. Therefore, wide band-gap II-VI ZnO quantum well (QW) structures have attracted much attention in terms of quantum efficiency because the exciton binding energy of ZnO QW is three times greater than that of GaN QW^36–40^. However, it has been difficult for the ZnO semiconductor to achieve a p-type doping, which is essential for device implementation^39^.

Cuprous halide semiconductors are intrinsically p-type direct band-gap semiconductors with large exciton binding energies. For example, CuI has a band-gap energy of 2.95 eV and an exciton binding energy of 62 meV. A high p-type mobility of 43.9 cm^2^ V^−1^ s^−1^ was reported for the polycrystalline CuI using the mineralizer for the hydrothermal method^26^. The lattice constant of cuprous halides is closely matched to that of Si^23^. However, growing single crystalline cuprous halides by epitaxial methods remains a challenge. In this study, CuI has been carefully chosen among cuprous halides, although CuCl has a better lattice match with Si due to the poor hydroscopic property of CuCl, which is detrimental to the device application. To date, only polycrystalline CuI thin films were grown by thermal evaporation and reactive sputtering technique^26^, but the crystal quality of the film was too poor to build optoelectronic devices. No work has ever been reported on the successful growth of the single crystalline CuI epitaxial film.

## Results

### Epitaxial layer growth of p-type CuI by molecular beam epitaxy

Here, we demonstrate the successful growth of a single crystalline CuI on Si (100) and sapphire (0001) substrates by molecular beam epitaxy (MBE). First, we studied the orientation and crystalline purity of the CuI crystal structure grown on a Si (100) substrate by high resolution X-ray diffraction (HR-XRD). Figure 1(b) shows an XRD 2θ scan of the CuI epitaxial layer on a Si substrate. It is interesting to note that only the (111) and (222) XRD peaks of CuI were observed along with the (200) and (400) Si peaks. Thin TEM samples were prepared using a dual beam focused ion beam (FIB) and were observed by transmission electron microscopy (TEM). A high resolution TEM image in Fig. 1(c) clearly shows the single crystalline structure of CuI growing on a Si substrate. Fast Fourier transform (FFT) patterns obtained from selected areas of the Si substrate and the CuI film indicate that Si (200) is parallel to CuI $(11\bar{1})$, indicating that the CuI epitaxial layer grows along the [111] direction on the Si (100) substrate. The epitaxial orientation relationship of (111) CuI//(100) Si can be comparable to the case^43^ of a (111) CdTe epitaxial layer grown on a GaAs (100) substrate having lattice constants of 0.646 nm and 0.565 nm, respectively. This growth behavior is due to the minimization of the interfacial potential energy with lattice mismatch of 14.3%^43^. The lattice mismatch of 11.4% between CuI (0.605 nm) and Si (0.543 nm) is large enough to follow the growth mode found in the CdTe/GaAs system.

We have grown numerous samples with different film thicknesses and found that the crystalline quality of CuI is almost independent of the sample thickness. Some typical growth data for CuI samples are summarized in Table S1 of the supplementary information (SI). The details of the epitaxial crystal growth of CuI on both Si (100) and sapphire (0001) substrates are given in section 1 of SI. The crystalline quality of the CuI samples we prepared was considered to be sufficiently high for applications of light-emitting devices with a less defect density of the order of 10^9^ cm^−2^ counted on surface images. However, currently, the defect density in best nitrides is in the order of 10^8^ cm^−2^ and more work would be needed to improve the crystal quality of CuI.

For the electrical characterization, we measured the electrical resistivity (ρ), Hall coefficients, carrier density (*n*_*b*_), and Hall mobility (*μ*) using van der Pauw geometry in an applied magnetic field of 1.0 Tesla. The input current of 1 μA was used to measure Hall voltage differences. The thickness-dependent resistivity and the Hall coefficient are shown in Table 1. The resistivity for CuI samples is in the range of 0.488∼2.084 Ω cm at 300 K. Although some deviations exist, as the average hole mobility and corresponding carrier density of CuI are $45.09\,c{m}^{2}{V}^{-1}{s}^{-1}$and $5.47\times {10}^{17}\,c{m}^{-3}$, respectively. These values compare favorably with those of p-GaN, as developed by Nakamura^44^ for early commercial light-emitting diodes (LEDs), whose resistivity, hole mobility and hole carrier concentration were $3\,\Omega \cdot cm$, $6\,c{m}^{2}{V}^{-1}{s}^{-1}$ and $3\times {10}^{17}c{m}^{-3}$, respectively. It should be noted that the mobility of CuI is seven times greater than that of GaN, which enhances hole injection rate into the active region; as a result, we can expect more efficient LEDs to have better thermal dissipation performance. In light of the entire numerical values essential for efficient LEDs, we believe that CuI will be a good alternative to GaN for optoelectronic devices.

**Table 1:** Thickness dependent resistivity and Hall measurement date of CuI.

CuI	Sample Number	#1	#2	#3	#4
	Thickness $[\mu m]$	0.283	0.596	0.820	1.340
	Resistivity $[\Omega \cdot cm]$	1.128	1.244	0.489	2.085
	Bulk carrier density $[c{m}^{-3}]$	1.06 × 10^18^	1.58 × 10^17^	9.05 × 10^17^	1.23 × 10^17^
	Hall mobility $[c{m}^{2}\cdot {V}^{-1}\cdot {s}^{-1}]$	110.27	31.69	14.12	24.32

### Enhanced photoluminescence of CuI epitaxial layer

**Figure 2:**
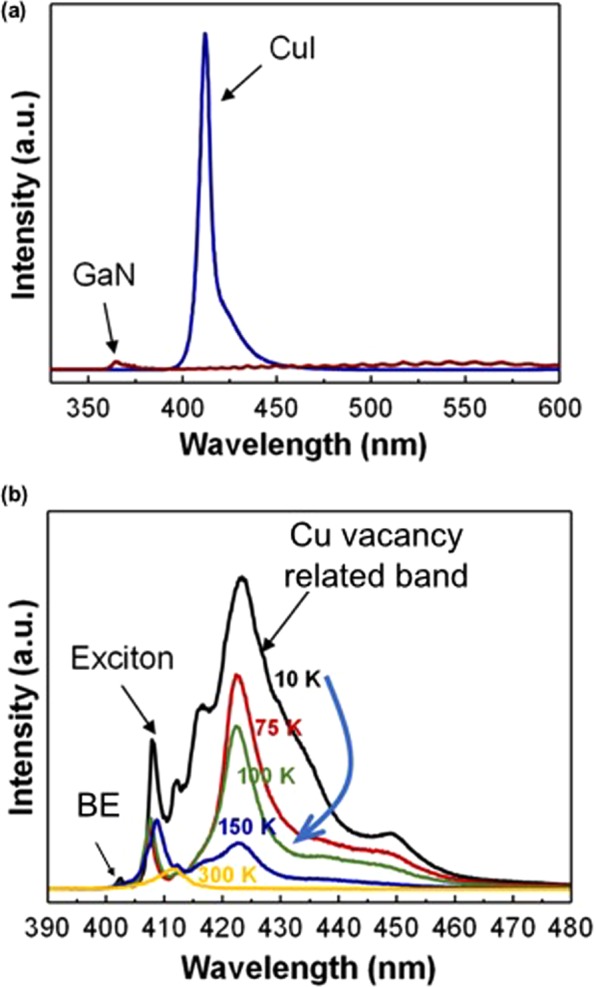
(**a**) Photoluminescence spectra for CuI sample #3 on Si substrate and for undoped GaN on sapphire substrate at room temperature. The wavelength of the PL peak corresponds to 415 nm for CuI and 365 nm for GaN, respectively. The thickness of the CuI samples varies from 283 nm to 1,340 nm (Table S1) and it is found that the PL peak position and intensity are almost independent of the sample’s thickness (Figure S3). (**b**) PL spectra for the CuI film sample #3 measured in the temperature range of 10 to 300 K. The PL spectra are dominated by the peak at 424 nm up to 150 K, which is believed to be caused by Cu vacancy transitions^45^.

Figure 2(a) shows the photoluminescence (PL) spectra of CuI on a Si (001) substrate and a commercially available undoped GaN that is grown on a sapphire substrate at room temperature for comparison. The PL peaks are located at 415 nm for CuI and 365 nm for GaN, respectively. The thickness of the CuI sample is 820 nm (Table S1). The PL peak position and intensity were found to be independent of the sample thickness (Fig. S3). The PL peak intensity of CuI is almost one order of magnitude higher than that of the commercially available undoped GaN samples grown on sapphire. These results are at least qualitatively consistent with the theoretical calculations discussed in the section 2 of SI. Fig. 2(b) shows the temperature dependence of the PL spectra for the CuI. The sample was inserted into a constant He-flow cryostat where the temperature remained constant during the PL measurement. The temperature was varied from 10 to 300 K. The PL spectra are dominated by the PL band at a wavelength of about 424 nm up to 150 K, which is presumably due to the Cu vacancy-related transitions^45^. The PL peaks around 409 and 402 nm are attributed to exciton-related transitions and band-edge related transitions, respectively. The energy difference between these two PL peaks is 52.8 meV, and this value is in good agreement with the calculated exciton binding energy of 62 meV. Considering the calculated biexciton binding energy (5.52 meV), both exciton and biexciton peaks were merged around 409 nm. With the strong PL intensity of the peak band associated with the Cu vacancies, we can explain the strong p-type of CuI in this article because Cu vacancies act as acceptors. From the temperature-dependent analysis, it is known that the main transition at room temperature (PL peak around 415 nm at Fig. 2(a)) is related to the exciton transition. The PL side wrap around 425 nm was connected to the Cu vacancy transition.

Observing the PL spectra of the exciton molecules indicates the high crystalline property of the CuI epitaxial layer. The generation of light from the active region of the optoelectronic device is described by $L(\omega )={L}_{0}\exp (G(J)l)$ where *L* is the light output, *G*(*J*) is the optical gain of the material, *J* is the injection current density and *l* is the length of the active region. The optical gain is related to the current density by $G(J)=\beta J-\alpha$ where *β* is the gain coefficient, which is proportional to PL and $\alpha$ is the loss of the material^46,47^. The results in Fig. 2 suggest that ${\beta }_{CuI} > {\beta }_{GaN}$ for bulk semiconductors.

### Optically pumped lasing of CuI epitaxial layer with vertical cavity

**Figure 3:**
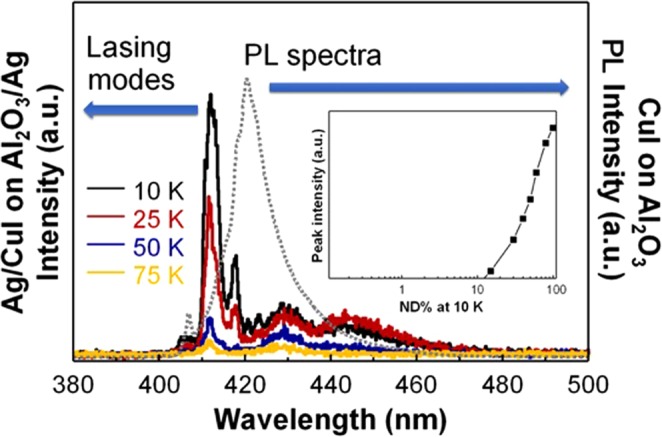
Optically pumped continuous wave (CW) lasing of the CuI epitaxial layer grown on sapphire with the vertical cavity at cryogenic temperature is observed. The lasing takes place at 10 K in a sample with a vertical cavity formed by the Ag coating on top of the CuI layer with a thickness of 1.15 $\mu m$ and the bottom of the sapphire substrate. The CW pumped lasing peak centered at around 412 nm with a narrow central mode is visible in this figure. The PL spectrum is also shown for comparison. The insert shows the log-log plot of the emission intensity as a function of the excitation power density denoted by the percentage of the neutral density (ND) filter employed to control the input power. We estimate the threshold power density to be approximately 250 W/cm^2^.

An optically pumped continuous wave (CW) lasing of the CuI epitaxial layer growing in the sapphire with the vertical cavity at cryogenic temperature is observed in Fig. 3. The cryogenic PL setup described above was used as a pumping source, and the lasing took place at 10 K in a sample with a vertical cavity formed by the Ag coating on the top of the 1.15 $\mu m$-thick CuI layer on the sapphire substrate and the Ag coating at the bottom of the sapphire substrate. The thickness of the Ag coating was 50 nm at the top of the CuI layer and 60 nm at the bottom of the sapphire substrate. This vertical cavity is partially transparent at a pump laser wavelength of 325 nm with the calculated reflection coefficient of 0.4, but is substantially reflective at 415 nm with the reflection coefficient of 0.86. The calculation was done using the propagator matrix for layered structures^48^. In this figure, it can be seen that the CW-pumped lasing peak, centered at 411.9 nm with a narrow central mode, is visible (solid lines). The PL spectrum is also shown for comparison (dotted line). It is interesting to note that the PL spectrum is dominated by the Cu vacancy transition (424 nm) at this temperature, while the lasing occurs at 411.9 nm, which is close to the exciton energy (409 nm). The optically pumped lasing mechanism is similar to that of the Ruby laser in which the levels ${R}_{1}$ and ${R}_{2}$ are analogous to the exciton and the Cu vacancy levels^49^. The results suggest that the loss in the cavity for the Cu vacancy transition is much greater than that of the exciton level in which population inversion occurs. The pump laser has a spot diameter of 1.6 mm and a maximum power density of $2.5\,{{\rm{KW}}/{\rm{cm}}}^{2}$. The insert shows a log-log plot of the emission intensity as a function of the excitation power density, expressed as a percentage of the neutral density (ND) filter employed to control the input power. We estimate that the threshold power density is approximately $250\,{{\rm{W}}/{\rm{cm}}}^{2}$. The absorption spectrum shown in Fig. S5 indicates that there is no preferred Fabry-Perot cavity mode and that the lasing is the result of the dominance of exciton transitions. The full width half maximum (FWHW) values of lasing peak are 2.801 nm, 2.954 nm and 2.730 nm for 10 K, 25 K and 50 K, respectively.

### Demonstration of a hybrid n-GaN/p-CuI blue LED

**Figure 4:**
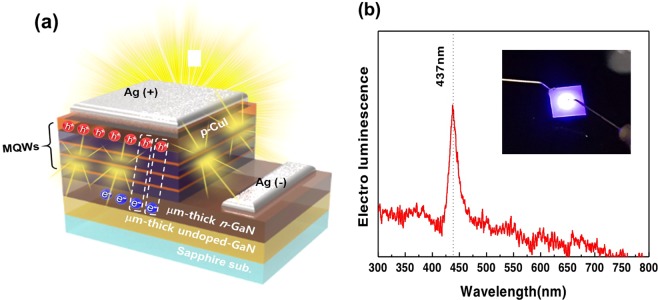
(**a**) Schematic diagram of a hybrid InGaN/AlGaN-CuI blue LED. (**b**) Electroluminescence (EL) spectrum of a hybrid blue LED. The epitaxial structure before the growth of the CuI layer consists of a sapphire substrate, an n-GaN layer, and InGaN/AlGaN multiple quantum wells. CuI layer was grown on top of the AlGaN barrier. The EL spectrum shows the peak at 437 nm.

As a validation of an intrinsic p-CuI, we have demonstrated a hybrid n-GaN/CuI blue LED in which the p-CuI epitaxial layer served as a hole injection layer as depicted in Fig. 4(a). The commercially available n-GaN epitaxial structure consists of a sapphire substrate, an n-GaN layer, and an InGaN/AlGaN multiple quantum wells (MQWs). A CuI epitaxial layer was grown on top of the AlGaN barrier to form a p-i-n junction. Structural analysis was performed to check the interface of the hybrid device. Figure S5 illustrates detailed data including the surface image, HR XRD, cross-sectional TEM image and schematics of the total structure. Figure 4(b) shows the electroluminescence (EL) spectrum of a hybrid blue LED. The peak of the EL spectrum is located at 437 nm. The inset shows the image of EL emitting from our hybrid LED. We have also shown a hybrid n-GaN/CuI ultraviolet (UV) LED in SI. Figure S6 shows the electroluminescence (EL) spectrum of a hybrid UV LED. The peak of the EL spectrum is located at 376 nm. Blue light emission shown in the inset is due to the addition of blue phosphorus to the surface of the hybrid UV LED.

Our goal for the future work is to achieve the high conductivity of CuI layer comparable to the best p-type conductivity of p-GaN which was reported with impurity resonant p-type doing^50,51^. P-type feature of CuI is due to Cu vacancies acting as acceptors. The conduction type is realized by the defects and the p-type conduction is unstable and uncontrollable. Therefore, the intrinsic semiconductor should be obtained firstly and the conduction type can be controlled using doping. We have done further experiments to see the possibility of obtaining the intrinsic CuI. In Table 2, we show the change of p-type bulk carrier density and the Hall mobility with the introduction of Zn doping. The bulk p-type carrier density is reduced from $9.05\times {10}^{17}c{m}^{-3}$ to $6.97\times {10}^{14}c{m}^{-3}$ and the hole mobility is increased from $14.12\,c{m}^{2}{V}^{-1}{s}^{-1}$ to $167.45\,c{m}^{2}{V}^{-1}{s}^{-1}$ for the sample #3 (without Zn doing) and the sample #6 (with Zn doing), respectively. Three orders of magnitude reduction of the p-type carrier density and two orders of magnitude increase of hole mobility is found with the incorporation of Zn doing. This indicates that the intrinsic CuI can be achieved by Zn doping. In addition, Figure S8 shows SEM images for the surface morphology and high resolution XRD 2θ scan of the Zn doped CuI thin film corresponding to samples #5 and #6 in the Table 2. Cell temperature variation of a Zn source in MBE is used for the flux change. Figure S8 shows that the good crystal quality is still achieved with Zn doping. We have also tried ion implantation of iodide to achieve the intrinsic condition. As a preliminary result, we have observed the bulk carrier density between $-2.552\times {10}^{13}c{m}^{-3}$ to $3.669\times {10}^{14}c{m}^{-3}$ for the iodide implantation energy of $350\,Kev$ and the flux density of $1\times {10}^{15}c{m}^{-2}$. The negative sign of the bulk carrier density indicates that iodide implanted sample becomes n-type. The electron (hole) mobility of the iodide implanted sample is $7.52\,c{m}^{2}{V}^{-1}{s}^{-1}$ and $9.66\,c{m}^{2}{V}^{-1}{s}^{-1}$, respectively. The mobility values of iodide implanted samples are much lower than those of Zn doping samples. This implies that the crystal structure may be damaged by the implantation process. Further studies will be conducted for the dopants and the ion implantation.

**Table 2:** Zn doping dependent resistivity and Hall measurement date of CuI.

CuI	Sample Number	#5	#6
	Thickness [*μm*]	0.854	0.947
	Resistivity $[\Omega \cdot cm]$	43.2	53.4
	Bulk carrier density $[c{m}^{-3}]$	1.67 × 10^15^	6.97 × 10^14^
	Hall mobility $[c{m}^{2}\cdot {V}^{-1}\cdot {s}^{-1}]$	86.13	167.45

In the commercial production of LEDs consisting of n- and p-GaN, the growth chamber must be cleaned after each growth of the p-GaN layer due to the memory effect of the Mg dopant, which could be a bottleneck of the entire process^44^. The hybrid LED demonstrated in this work will provide an excellent approach in overcoming the process hurdle. In addition, the low growth temperature of CuI at around 200 °C makes it compatible with other types of LEDs, such as organic light-emitting diode. Potential drawback of the hybrid LED with p-CuI would be the need of the second growth process after MOCVD growth.

## Discussion

In this report, we have successfully demonstrated the single-crystal epitaxial growth of CuI. The crystalline quality has proven to be sufficient for photonic device applications. The epitaxial CuI is an intrinsic p-type and its electrical properties are superior to early developed p-GaN for commercial LEDs. PL peak intensities of CuI at room temperature were found to be an order of magnitude greater than that of commercially available undoped GaN sample on sapphire. CW lasing of CuI on sapphire is realized at low temperature for the vertical cavity structure. Enhanced PL and CW lasing can also be considered as a measure of an excellent crystal quality of the epitaxial CuI layer. We have also presented a hybrid InGaN/AlGaN MQWs -CuI blue LED with a CuI layer that served as a p-injection layer instead of p-GaN, which may have significant industrial impacts in terms of greatly reducing the number of processes by eliminating the cleaning steps in each growth of p-GaN doped with Mg. In summary, our results propose a new material based on cuprous halides for highly efficient optoelectronic devices compatible with Si and GaN technologies.

## Methods

Detailed experimental processes are provided in the Supporting Information.

## Supplementary information

Supplementary Information.

## Data Availability

All data generated or analysed during this study are included in this article (and its supplementary information files). The data that support the finding of this study are also available from the corresponding author upon reasonable request.
