# Supplementary material for: Intrinsically p-type cuprous iodide semiconductor for hybrid light-emitting diodes
Source: Sci Rep. 2020 Mar 4;10:3995. doi: 10.1038/s41598-020-61021-2 (PMC7055318; doi:10.1038/s41598-020-61021-2)
Supplement: Supplementary file 1 — Supplementary Information. [file 41598_2020_61021_MOESM1_ESM.docx]

**Supplementary Information for**

Intrinsically p-type cuprous iodide semiconductor

for hybrid light-emitting diodes

D. Ahn^1,2,3*^, J. D. Song^4*^, S. S. Kang^4,5^, J. Y. Lim^1^, S. H. Yang^1^, S. Ko^1^, S. H. Park^6^, S. J. Park^7^, D. S. Kim^8^, H. J. Chang^4^ and Joonyeon Chang^4*^

^1^Peta Lux Inc., 3F TLi Building, 12 Yanghyeon-ro, 405 beon-gil, Jungwon-gu, Seongnam-si, Gyeonggi-do 13438, Republic of Korea

^2^Department of Electrical and Computer Engineering and Center for Quantum Information Processing, University of Seoul, 163 Seoulsiripdae-ro, Dongdaemun-gu, Seoul 02504, Republic of Korea

^3^*Physics Department, Charles E. Schmidt College of Science, Florida Atlantic University, 777 Glades Road, Boca Raton, FL 33431-0991, USA*

^4^ *Post-Silicon Semiconductor Institute*, *Korea Institute of Science and Technology*

Hwarang-ro 14 gil, Seoungbuk-ku, Seoul 02792, Republic of Korea

^5^ *Department of* *Physics, Kyung Hee University, 26 Kyungheedae-ro, Dongdaemun-gu, Seoul 02447, Republic of Korea*

*^6^Electronics Department, Catholic University of Daegu, 13 Hayang-Ro, Hayang-Eup, Gyeongsan-si, Gyeongbuk, 38430 Republic of Korea*

*^7^WONIK IPS, 75 Jinwisandan-ro, Jinwi-myeon, Pyeingtaek-si, Gyeonggi-do 17709, Republic of Korea*

^8^TLi Inc., 10F TLi Building, 12 Yanghyeon-ro, 405 beon-gil, Jungwon-gu, Seongnam-si, Gyeonggi-do 13438, Republic of Korea

*To whom correspondence should be addressed;

E-mail: [dahn@uos.ac.kr](mailto:dahn@uos.ac.kr); [jdsong@kist.re.kr](mailto:jdsong@kist.re.kr); [presto@kist.re.kr](mailto:presto@kist.re.kr)

**In this supplementary information of our article, we describe the experimental methods and the theoretical model in detail. On the experimental side, the demonstration of the single-crystal epitaxial growth of Cul on Si (100) and Sapphire (0001) substrates is described. Additional materials for photoluminescence measurements, lasing from the vertical cavity and InGaN/CuI hybrid LED are provided. Theoretical studies on band structures and spontaneous emission spectra of I-VII zinc-blende CuI bulk semiconductors in the short-wavelength spectral range are also described.**

1. **Experimental results for Cul epitaxial growth on Si and sapphire substrates.**

We have succeeded in growing a single crystalline CuI on Si (100) and on sapphire (0001) by Molecular Beam Epitaxy (MBE). All the samples were grown in a home-modified MBE system originally supplied by Riber SA with ion getter pumps (600 L/s). The chamber was modified with materials resistant to corrosive environments. The pressure of the main chamber before growth is approximately 1.0 × 10^-10^ Torr. Powder-typed CuI (anhydrous, 99.995% trace metals basis) supplied by Sigma-Aldrich company was used as a CuI source and was evaporated using standard 60 cc K-cells. The area of Si substrates used for the deposition of CuI thin films was 18 mm × 18 mm in size. The Si substrates were cleaned with acetone, methanol and deionized water in an ultrasonic bath for 1~2 min each. The native oxide for the Si substrate was removed in the main chamber above 800°C. The growth temperature of CuI is in the range of 150-200°C, and the pressure during growth is in the range of 1.0~30.0 × 10^-7^ Torr.

We first studied the orientation and phase purity of the CuI crystal structure grown on Si substrate by high resolution X-ray diffraction (HR-XRD) using an ATX-G. Figure S1(a) shows the XRD 2θ scan of CuI thin film samples grown on Si substrate. The XRD peaks of CuI correspond to the (111) and (222) plane. It is interesting to note that only the (111) and (222) peaks are observed in the CuI sample that grows on the Si (100) substrate and that the orientation and crystalline purity are almost independent of the sample thickness, although the area of a typical grain becomes larger as thickness is enlarged. The growth data for CuI samples are summarized in Table S1. In Figure S1(b), we show the scanning electron microscopy (SEM) images for the surface morphology of the CuI samples. The SEM image shows triangular shaped grain corresponding to the (111) oriented γ –CuI, and the typical area of each grain is enlarged as the thickness is larger, which is attributed to the combination of crystal grains.

**Table S1.** Growth information on CuI samples

| Sample Number | #1 | #2 | #3 | #4 |
| --- | --- | --- | --- | --- |
| Growing time (h) | 1.0 | 2.0 | 3.0 | 5.0 |
| Thickness (μm) | 0.283 | 0.596 | 0.820 | 1.340 |
| Growth rate (μm/h) | 0.283 | 0.298 | 0.273 | 0.268 |

We also studied the orientation and phase purity of the CuI crystal structure grown on sapphire substrate by HR-XRD using an ATX-G. Figure S2 shows the XRD 2θ scan of CuI thin film samples grown on Si substrate. The XRD peaks of CuI correspond to the (111) and (222) planes.

Photoluminescence (PL) spectra from 10 K to 300 K were obtained using a 325nm He–Cd laser as excitation light with an optical power of 5 mW in the sample. The sample emission was focused onto a 300 mm focal length spectrograph and detected by a TE cooled CCD detector (2000 x 256 pixels, Back-illuminated type). All PL spectra were corrected taking into account the spectral response of the system.

Figure S3 shows series of PL spectra of four CuI samples with different thicknesses grown on Si (100) substrate and compared with a commercially available un-doped GaN grown on sapphire substrate at room temperature (300K). The PL peak positions of these samples are 415 nm for CuI and 365 nm for GaN, respectively. The thickness of the CuI samples varied from 283 nm to 1,340 nm (Table S1), and the PL peak position and intensity were found to be almost independent of the sample thickness.

Figure S4 shows the absorption spectra of a 50 nm-thick Ag/CuI/Sapphire/60nm-thick Ag vertical cavity structure. It is obvious that there is no preferred Fabry-Perot resonance mode for this cavity within the range of 400 and 440 nm. This reflects that the stimulated light emission outside the vertical cavity is not attributed to the cavity structures rather than selection within CuI. This was measured by a Lambda 750 UV/VIS/NIR spectrophotometer and manufactured by PerkinElmer.

In Figure S5, the left side shows the surface SEM, XRD, cross-sectional TEM and schematic of an InGaN/AlGaN MQWs/CuI hybrid LED, where the CuI layer is grown on top of the AlGaN quantum-well barrier for use as p-injection layer. The surface SEM image shows a grain of triangular shape corresponding to the (111) oriented γ –CuI. The XRD peaks of CuI are related to the (111) and (222) planes. The cross-sectional TEM and schematic of the hybrid structure were compared at the bottom of Figure S5 after contact metal deposition. It shows that CuI grows directly on the AlGaN MQW barrier. The growth of the InGaN/AlGaN MQWs LED epi-layer stops just above the MQWs and is supplied by the local MOCVD-based LED company. Recently, it is found that adding additional cladding layer of approximately 40 nm-thick GaN layer above AlGaN MQW below CuI layer enhances the electrical property of the LED. It is attributed to the reduction of the barrier between the MQW and the CuI layer.

Figure S6 shows electroluminescence (EL) spectrum of a hybrid UV LED. The epitaxial structure before the growth of the CuI layer consists of a sapphire substrate, an n-GaN layer, and InGaN/AlGaN multiple quantum wells. A 30 nm of CuI layer was grown on top of the AlGaN barrier. The EL spectrum shows the peak at 376 nm.

The defects shown in the SEM surface image of Figure S5 are related to the defects found in the red circle of the cross-sectional TEM image of Figure S5, resulting in multiple contacts after metal deposition.

Figure S7 shows the PL spectrum of an InGaN/CuI hybrid LED structure for different CuI layer thicknesses of 30 nm and 300 nm. The PL spectrum was found to be dominated by InGaN QWs for a thin CuI layer (30 nm), but was used for the thick CuI layer (300 nm) by CuI excitons as expected. Figure S7 displays the relative strength of the PL peaks from the InGaN/AlGaN MQWs and the upper CuI layer. It is interesting to note that the PL peak is dominated by the InGaN/AlGaN MQW for the thin CuI epitaxial layer (30 nm), but the CuI PL peak becomes dominant for the thick CuI epitaxial layer (300 nm).

Figure S8 shows the scanning electron microscopy (SEM) for the surface morphology and high resolution X-ray diffraction (XRD) 2θ scan of the Zn doped CuI thin film samples #5 and #6 of the Table 2.

1. **Theoretical Model**

Here, the band structures and spontaneous emission spectra of cuprous halides zinc-blende CuI semiconductor in the short-wavelength spectral range are theoretically studied by the multiband effective mass theory and the optical model with many-body effects. These results are also compared with those of conventional short-wavelength wurtzite GaN bulk semiconductor.

1. (001)-oriented zinc-blende 6x6 Luttinger-Khon Hamiltonian

Based on the theories of Luttinger–Kohn and Bir–Pikus^1-3^, the valence band structure of a strained zinc-blende semiconductor can be described as^4,5^

where

$$P_{\varepsilon}=-a_{v}(\varepsilon_{xx}+\varepsilon_{yy}+\varepsilon_{zz}),$$

$$Q_{\varepsilon}=-\frac{b}{2}(\varepsilon_{xx}+\varepsilon_{yy}-2\varepsilon_{zz}),$$

and for $i,j=x,y,z$is a symmetric strain tensor; $\gamma_{1},\gamma_{2}$, and $\gamma_{3}$ are the Luttinger parameters; $a_{v},b$, and $d$are the Bir-Pikus deformation potentials; $\Delta$ is the spin-orbit split-off energy; $m_{0}$is the free electron mass, is Planck's constant divided by $2\pi$, and $k_{i}$is the wave vector. The superscript † means taking both transpose (~) and complex conjugate (*). Here, we neglect the strains for bulk semiconductors. The basis for the Hamiltonian are:

$$\begin{matrix} |u_{1}\rangle=-\frac{1}{\sqrt{2}}|\left(X+iY\right)\uparrow\rangle, \\ |u_{2}\rangle=\frac{1}{\sqrt{2}}|\left(X-iY\right)\uparrow\rangle, \\ |u_{3}\rangle=|Z\uparrow\rangle, \\ |u_{4}\rangle=\frac{1}{\sqrt{2}}|\left(X-iY\right)\downarrow\rangle, \\ |u_{5}\rangle=-\frac{1}{\sqrt{2}}|\left(X+iY\right)\downarrow\rangle, \\ |u_{6}\rangle=|Z\downarrow\rangle. \end{matrix}$$

1. (0001)-oriented wurtzite 6x6 Luttinger-Khon Hamiltonian

The c-plane Hamiltonian for the valence band of the (0001)-oriented wurtzite semiconductor is given as^5,6^

$$H_{11}=\Delta_{1}+\Delta_{2}+\lambda+\theta,$$

$$H_{22}=\Delta_{1}-\Delta_{2}+\lambda+\theta,$$

$$H_{33}=\lambda+\lambda{}_{\varepsilon},$$

$\lambda_{\varepsilon}=D_{1}\varepsilon_{zz}+D_{2}(\varepsilon_{xx}+\varepsilon_{yy}),$ $\theta_{\varepsilon}=D_{3}\varepsilon_{zz}+D_{4}(\varepsilon_{xx}+\varepsilon_{yy})$.

and the $A_{i}$’s are the effective-mass parameters of the valence band analogous to the Luttinger parameters for the zinc-blende semiconductors, the $D_{i}$’s are the deformation potentials for wurtzite semiconductors, $\Delta_{1}$is the crystal-field split energy, and $\Delta_{2}$and $\Delta_{3}$are the spin-orbit interaction energies.

The simplest non-Markovian quantum kinetics^7^ is the Gaussian line-shape function, which is connected with memory effects in the system–reservoir interaction. The spontaneous emission spectra with many-body effects, including the effects of anisotropy on the valence band dispersion are given as^7,8^

$g_{sp}(\omega)=\sqrt{\frac{\mu_{o}}{\varepsilon}}\left(\frac{e^{2}}{m_{o}^{2}\omega}\right)\frac{2}{(2\pi)^{3}}\int_{0}^{\infty} dk_{x}dk_{y}dk_{z}\left|M_{cv}(k)\right|^{2}f_{c}(1-f_{v})\mathrm{Re}\left\{\frac{L(\omega,k)}{1-Q(k)}\right\},$

where $\omega$ is the angular frequency, $\mu_{o}$ is the vacuum permeability, $\varepsilon$ is the dielectric constant, $e$ is the charge of an electron, $m_{o}$ is the free electron mass, $k$ is the magnitude of the wave vector, $\left|M_{cv}(k)\right|^{2}$ is the momentum matrix element, $f_{c}$and $f_{v}$are the Fermi functions for occupation probability by the electrons in the conduction band states and the valence band states, respectively. The Gaussian line shape function renormalized with many-body effects is given as^7,8^

Here, is the transition energy between electrons and holes, where $E_{g}$ is the band gap of the material, and $\Delta E_{BGR}$ is the band-gap renormalization, respectively. The correlation time $\tau_{c}$ is related to the non-Markovian enhancement of the optical gain^7^ and is assumed to be constant. The $\tau{}_{in}$and the $\tau_{c}$ used in the calculation are 25 and 10 fs, respectively. The Coulomb enhancement factor is given as^7,8^

,

,

where .

The material parameters for wurtzite GaN used in the calculation were taken from Ref. 5 and references therein. All parameters for CuI used in the calculation are from reference 9.

Figure S9 shows bulk valence band structures of unstrained (a) (0001)-oriented wurtzite GaN and (b) (001)-oriented zinc-blende CuI. The valence band energy is plotted as a function of $k_{x}(1/\mathop{\text{A}}\limits^{o} )$. The wurtzite structure shows a crystal-field split-off hole (CH) band below the heavy-hole (HH) and light-hole (LH) bands, the number of which is determined by the crystal-field splitting due to its hexagonal symmetry, while the spin-orbit interaction removes the degeneracy between the HH and LH bands. Similarly, the zinc-blende structures also show the degeneracy between the HH and LH bands at the zone center. On the other hand, there is a spin-orbit split-off band in the case of a CuI semiconductor below the HH and LH bands by$\Delta_{so}$.

Figure S10 shows the spontaneous emission spectra of unstrained (100)-oriented zinc-blende CuI, and (0001)-oriented wurtzite GaN. The spontaneous emission spectra are calculated at a carrier density of N=1x10cm. The spontaneous emission peak of (100)-oriented zinc-blende CuI is greater than that of the (0001)-oriented wurtzite GaN, and the transition wavelength of the former is longer than that of the latter. This can be explained by the fact that the quasi-Fermi level (-23 meV) in the valence band of the former is much larger than that (-49 meV) of the latter. This means that the quasi-Fermi level in the valence band is relatively important in determining the light intensity.

Figure S11 shows the optical matrix elements of unstrained (a) (0001)-oriented wurtzite GaN, (b) and (c) (001)-oriented zinc-blende CuI. The optical matrix element of the (100)-oriented zinc-blende CuI is smaller than that of the (0001)-oriented wurtzite GaN. This is mainly due to the fact that the electron effective mass (0.3$m_{o}$) of the (100)-oriented zinc-blende CuI is larger than that (0.2$m_{o}$) of the (0001)-oriented wurtzite GaN. The larger electron effective mass of CuI results in a smaller bulk momentum matrix element.

**References**

1. J. M. Luttinger and W. Kohn, Phy. Rev. 97, 869 (1955).
2. J. M. Luttinger, Phy. Rev. 102, 1030 (1956).
3. G. L. Bir and G. E. Pikus, Symmetry and Strain-Indeced Effects in Semiconductor (Wiley, New York, 1974).
4. C. Y.-P Chao and S. L. Chuang, Phy. Rev.B 46, 4110 (1992).
5. S.-H. Park and S.-L. Chuang, J. Appl. Phys. 87, 353 (2000).
6. S. L. Chuang and C. S. Chang, Phys. Rev. B 54, 2491 (1996).
7. D. Ahn, Prog. Quantum Electron. 21, 249 (1997).
8. S. H. Park, S. L. Chuang, and D. Ahn, Semicond. Sci. Technol. 15, 203 (2000).
9. D. Ahn and S.-L. Chuang, Appl. Phys. Lett. 102, 121114 (2013).
10. Nishida, N., Saiki, K., & Koma, A. Hetroepitaxy of CuCl on GaAs and Si substrates. *Surf. Sci.* **324**, 149-158 (1995).
11. C. Yang, M. Kneiß, F.-L. Schein, M. Lorenz, M. Grundmann, Sci. Rep. 6, 21937 (2016)


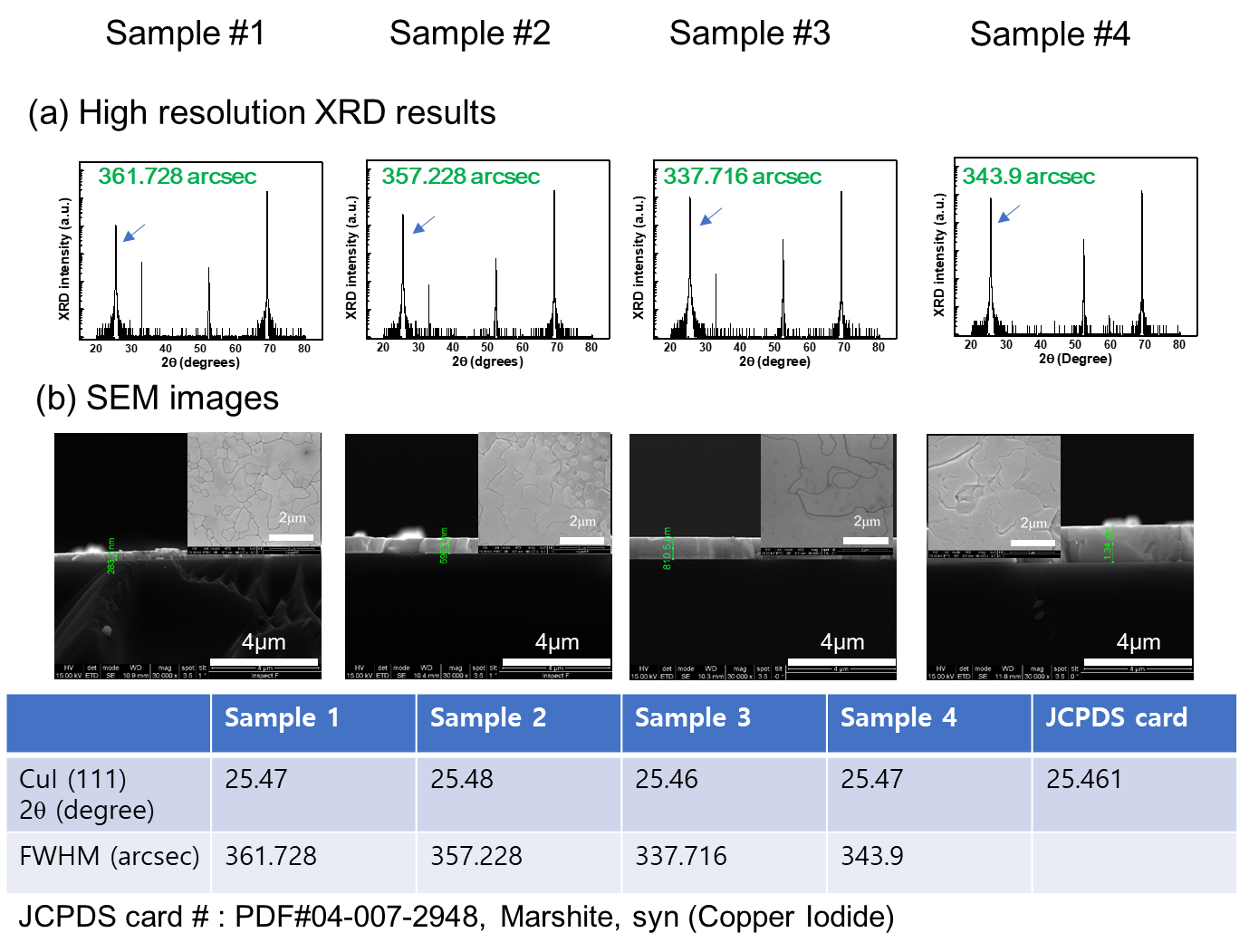


Figure S1. (a) High resolution X-ray diffraction (XRD) 2θ scan of the CuI thin film samples grown on the Si (100) substrate. The XRD peaks of CuI correspond to the (111) and (222) planes. (b) The results of scanning electron microscopy (SEM) for the surface morphology of the CuI samples. The SEM image shows the grain of triangular shape corresponding to (111) oriented $\gamma$-CuI.

Figure S2. High resolution X-ray diffraction (XRD) 2θ scan of the CuI thin film samples grown on the sapphire (0001) substrate. The XRD peaks of CuI correspond to the (111) and (222) planes, respectively.

Figure S3. Series of photoluminescence spectra at room temperature of four CuI samples with different thicknesses grown on the Si (100) substrate are shown and compared with a commercially available un-doped GaN grown on the sapphire substrate.

Figure S4. Absorption spectrum of the Ag/CuI/Saphirre/Ag vertical cavity structure


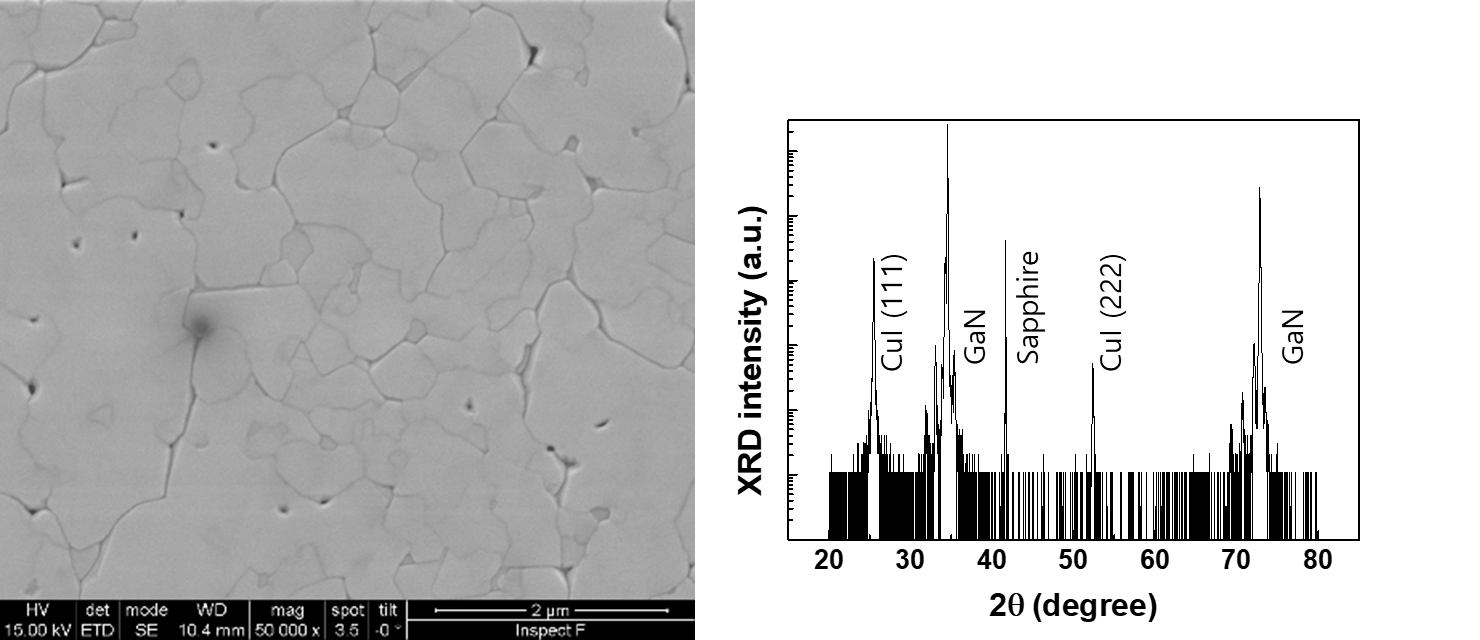


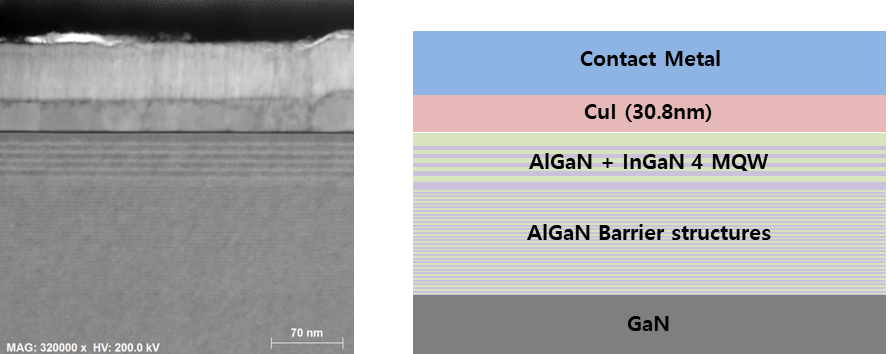


Figure S5. SEM surface image, high resolution X-ray diffraction 2θ scan before metal deposition and cross-sectional TEM image and the schematic of an InGaN/AlGaN/CuI hybrid LED after metal deposition. The CuI layer was grown on top of the AlGaN quantum-well barrier and used as p-injection layer.


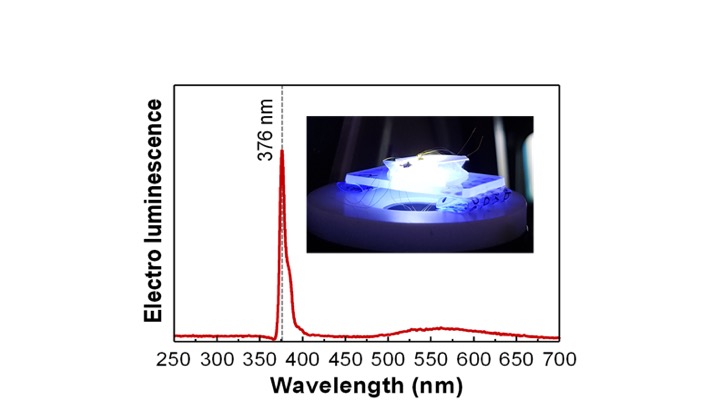


Figure S6. Electroluminescence (EL) spectrum of a hybrid UV LED. The epitaxial structure before the growth of the CuI layer consists of a sapphire substrate, an n-GaN layer, and InGaN/AlGaN multiple quantum wells. A 30 nm of CuI layer was grown on top of the AlGaN barrier. The EL spectrum shows the peak at 376 nm.


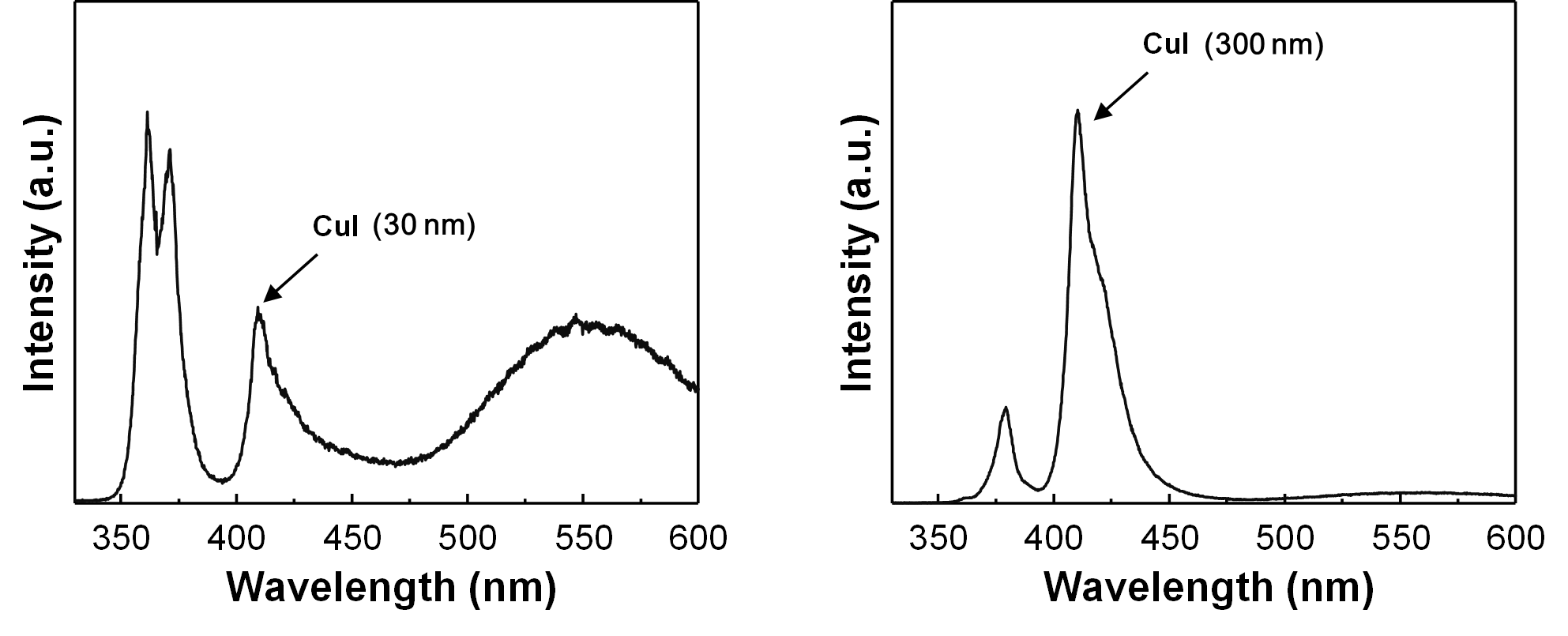


Figure S7. The photoluminescence spectra of the InGaN/CuI hybrid LED structure with different CuI layer thicknesses of 30 nm and 200 nm are shown.


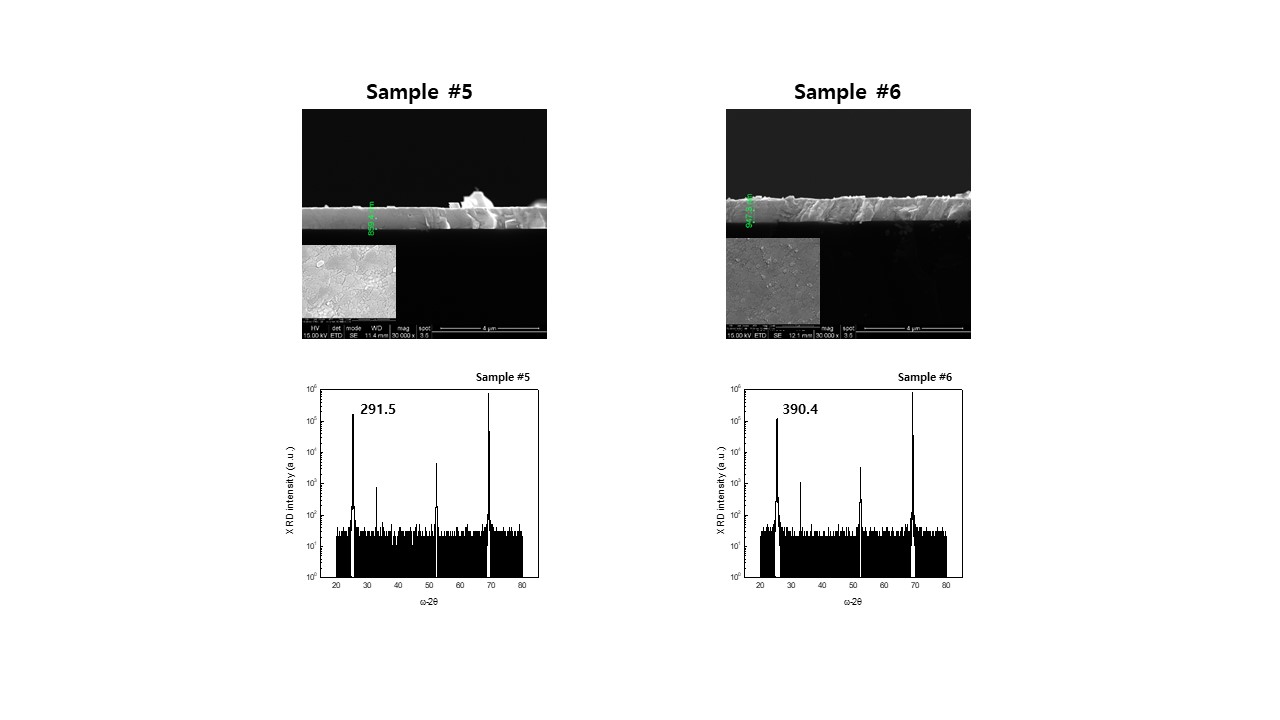


Figure S8 Scanning electron microscopy (SEM) for the surface morphology and high resolution X-ray diffraction (XRD) 2θ scan of the Zn doped CuI thin film samples #5 and #6 of the Table 2.


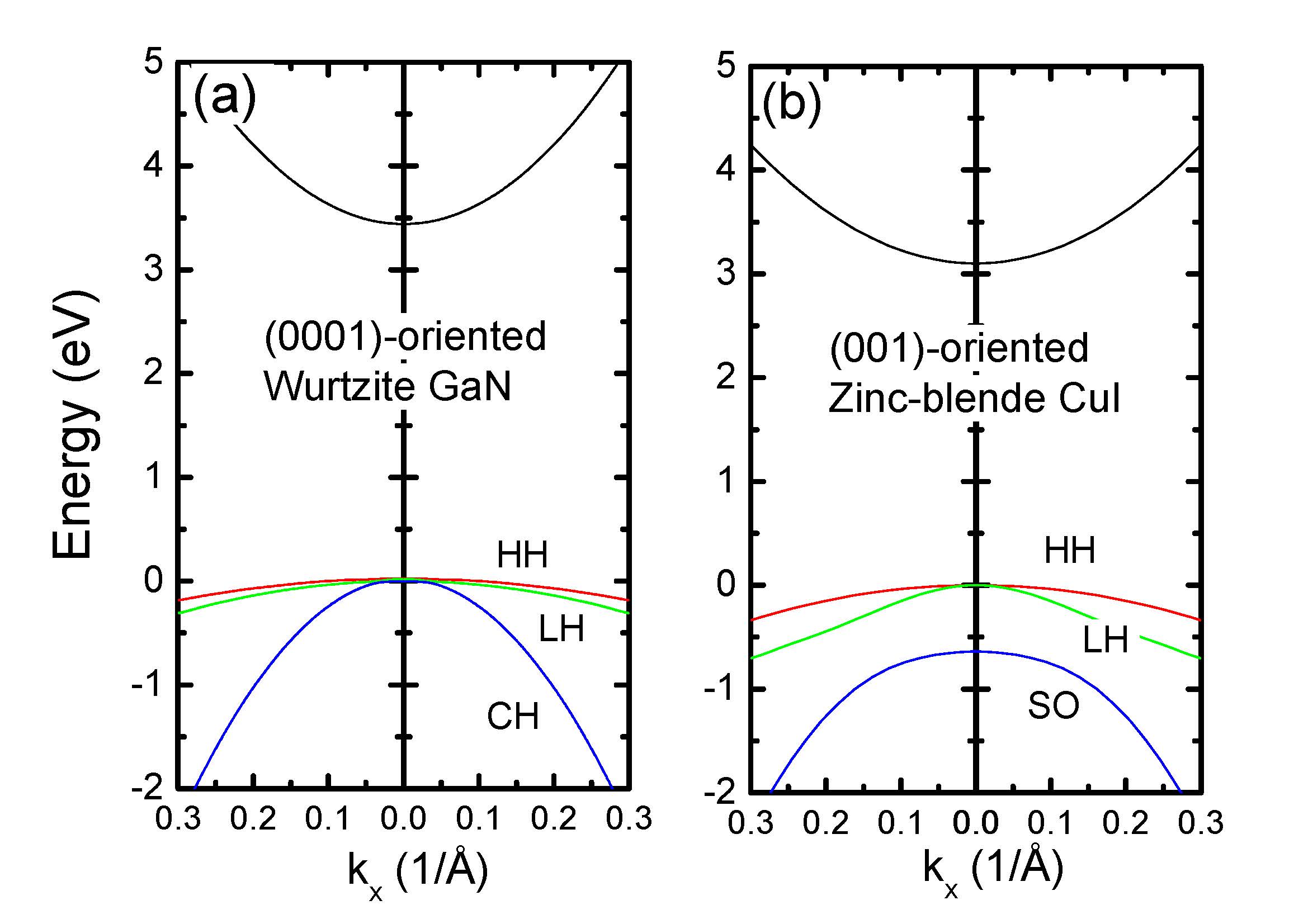


Figure S9. Bulk valence band structures of unstrained (a) (0001)-oriented wurtzite GaN and (b) (100)-oriented zinc-blende CuI are plotted as a function of $k_{x}(1/\mathop{\text{A}}\limits^{o} )$.


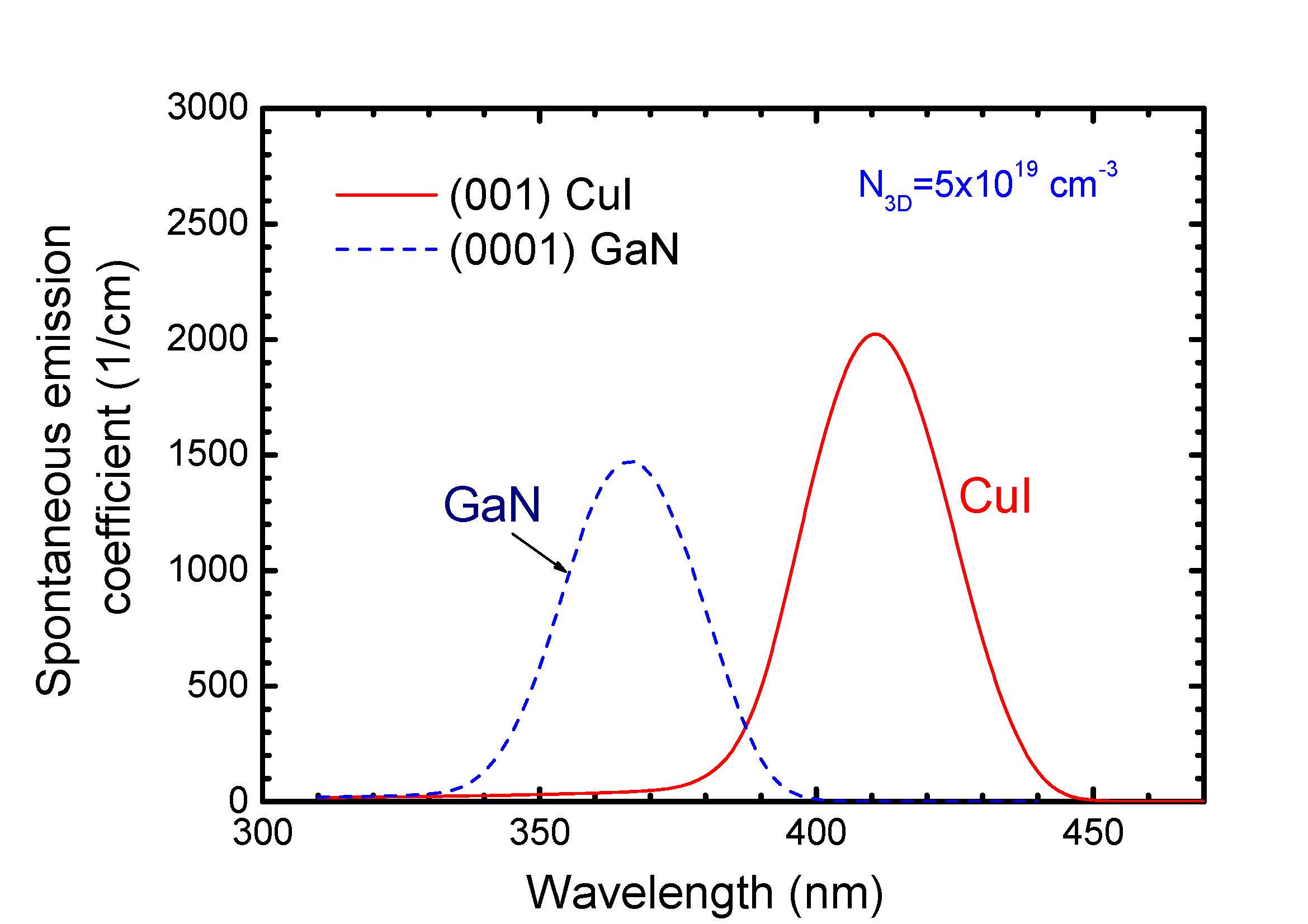


Figure S10. Spontaneous emission spectra of unstrained (001)-oriented zinc-blende CuI, and (0001)-oriented wurtzite GaN.


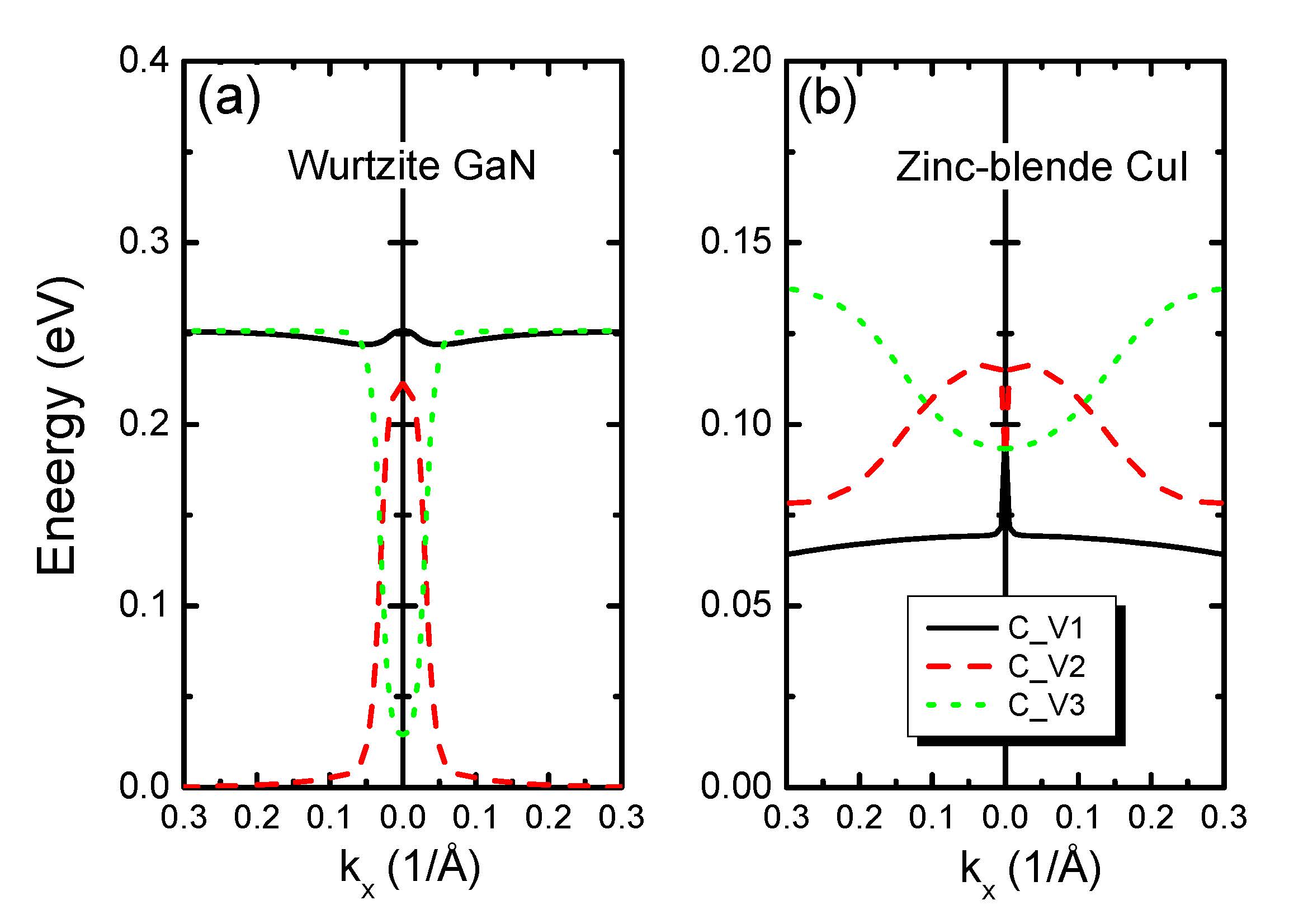


Figure S11. Optical matrix elements of unstrained (a) (0001)-oriented wurtzite GaN, (b) and (c) (100)-oriented zinc-blende CuI are shown. The optical matrix element of the (100)-oriented zinc-blende CuI is smaller than that of the (0001)-oriented wurtzite GaN.
